# Prognostic Correlation of an Autophagy-Related Gene Signature in Patients with Head and Neck Squamous Cell Carcinoma

**DOI:** 10.1155/2020/7397132

**Published:** 2020-12-28

**Authors:** Cai Yang, Hongxiang Mei, Liang Peng, Fulin Jiang, Bingjie Xie, Juan Li

**Affiliations:** ^1^Department of Orthodontics, West China Hospital of Stomatology, West China School of Stomatology, Sichuan University, State Key Laboratory of Oral Diseases, Chengdu 610041, China; ^2^Intelligent Information Technologies and Applications Lab (IIT), School of Information and Software Engineering, University of Electronic Science and Technology of China, Chengdu 610041, China

## Abstract

Considerable evidence indicates that autophagy plays a vital role in the biological processes of various cancers. The aim of this study is to evaluate the prognostic value of autophagy-related genes in patients with head and neck squamous cell carcinoma (HNSCC). Transcriptome expression profiles and clinical data acquired from The Cancer Genome Atlas (TCGA) database were analyzed by Cox proportional hazards model and Kaplan–Meier survival analysis to screen autophagy-related prognostic genes that were significantly correlated with HNSCC patients' overall survival. Functional enrichment analyses were performed to explore biological functions of differentially expressed autophagy-related genes (ARGs) identified in HNSCC patients. Six ARGs (EGFR, HSPB8, PRKN, CDKN2A, FADD, and ITGA3) identified with significantly prognostic values for HNSCC were used to construct a risk signature that could stratify patients into the high-risk and low-risk groups. This signature demonstrated great value in predicting prognosis for HNSCC patients and was indicated as an independent prognostic factor in terms of clinicopathological characteristics (sex, age, clinical stage, histological grade, anatomic subdivision, alcohol history, smoking status, HPV status, and mutational status of the samples). The prognostic signature was also validated by data from the Gene Expression Omnibus (GEO) database and International Cancer Genome Consortium (ICGC). In conclusion, this study provides a novel autophagy-related gene signature for predicting prognosis of HNSCC patients and gives molecular insights of autophagy in HNSCC.

## 1. Introduction

Head and neck squamous cell carcinomas include several types of malignancies from the oral cavity, pharynx, and larynx. Each year, over 600,000 cases are diagnosed worldwide, making HNSCC the sixth most prevalent cancer [1, 2]. Studies have shown the risk factors related to HNSCC are tobacco use, alcohol consumption, and human papillomavirus (HPV) infection. The typical treatments include surgery, radiotherapy, and chemotherapy. However, the mortality rate of HNSCC remains high, which results in 380,000 death annually mainly because of local recurrence and distant metastasis [3, 4]. The current plight is partly on account of the lack of accurate and reliable biomarkers for prognosis prediction in the early stage of this disease [5]. Therefore, it is quite essential to explore an effective and specific signature to help severity assessment and guide decision-making in clinical practice.

Autophagy is an important process which selects and degrades dysfunctional organelles, microbes, and proteins by lysosomes to maintain cellular homeostasis and sustain metabolism. Aberrant expression of autophagy-related genes (ARGs) is closely related to multiple diseases, especially neurodegenerative disorder, inflammatory abnormality, and cancer [6]. Autophagy plays a multifaceted role in tumor initiation and progression in respect of microenvironment stress, nutritional supplement, and immune status [7, 8]. Research suggests that autophagy-related mechanism is an optimistic target for future cancer therapy [9]; thus, exploring the valuable knowledge of autophagy is in urgent need and can offer huge potential in long term.

In this study, we comprehensively analyzed the correlation between expression profiles of ARGs and clinical features of HNSCC patients from The Cancer Genome Atlas (TCGA) database and constructed a 6-ARG risk signature. This model was identified to be an independent prognostic signature for HNSCC patients. Functional analysis was applied to reveal more information of ARGs. These findings could provide novel biomarkers for predicting the survival of HNSCC patients and give new insights in personalized therapy.

## 2. Materials and Methods

### 2.1. Data Acquisition and Preparation

RNA sequencing data of 545 HNSCC specimens, simple nucleotide variation data of 506 HNSCC specimens, and corresponding clinical information of 528 patients were downloaded from TCGA database (https://cancergenome.nih.gov/). A total of 232 ARGs were obtained from the Human Autophagy Database (HADb; http://autophagy.lu/clustering/index.html). We extracted autophagy-related gene expression data using Practical Extraction and Report Language (Perl; https://www.perl.org/). The clinical data was combined with corresponding gene expression profiles by unique ID of the patients. Samples without complete survival information or with overall survival time less than 7 days were excluded. Consequently, the data of 495 patients were retained for gene signature construction and further analyses. Three independent microarray HNSCC cohorts obtained from the GEO database (GSE41613, *n* = 97; GSE117973, *n* = 77) and ICGC (ORCA-IN, *n* = 40) were used as the testing groups. Gene expression data from the GSE117973 dataset was normalized using the zero-mean normalization method implemented in the Sklearn library with Python programming language [10].

### 2.2. Identification of Differentially Expressed ARGs and Functional Annotation

R programming language (version 3.6.2) was utilized as data analysis and plotting tool throughout this research. The R package limma was used to select differentially expressed ARGs between HNSCC and nontumor samples. After normalization by the formula log2(*x* + 1) transformed, 38 differentially expressed ARGs were identified with log fold change (FC) > 1 or <-1 and adjusted *p* value < 0.05. To investigate biological functions of the selected ARGs, gene ontology (GO) enrichment analysis and Kyoto Encyclopedia of Genes and Genomes (KEGG) pathway analysis were performed by R software and *p* < 0.05 was considered to be of statistically significant difference. The GOplot package was applied to visualize the enrichment terms.

### 2.3. Construction of the Prognostic Signature

Univariate Cox regression analysis was performed in the training set (TCGA cohort) to evaluate the association between differentially expressed ARGs and overall survival (OS) of HNSCC patients. ARGs with *p* value < 0.05 were considered to be statistically significant for OS and were identified as candidate genes. In order to minimize partial likelihood deviance and prevent overfitting of the model, we performed the least absolute shrinkage and selection operator (LASSO) Cox regression analysis and selected the most effective prognostic ARGs. The selected ARGs were subjected to the multivariate Cox regression analysis to further screen genes which were capable of predicting the prognosis independently. Subsequently, a risk model composed of several ARGs was established using glmnet and survival R packages.

### 2.4. Validation of ARG-Based Risk Model

The risk score of each patient was developed by a linear combination of the expression level of genes multiplied regression coefficients calculated by the Cox regression model. HNSCC patients were assigned into the high-risk or low-risk groups according to corresponding risk scores, using the median score as a cutting-off point. A Kaplan–Meier survival curve was plotted to estimate the difference between the two groups using the log-rank test. Meanwhile, the time-dependent receiver operating characteristic (ROC) analysis was employed to evaluate the efficiency of the prognostic signature using the survivalROC package, and the respective area under the ROC curve (AUC) was assessed for predicting accuracy.

To verify if the prognostic ARGs signature could be an independent indicator for predicting the OS of patients with HNSCC, the multivariate Cox regression analysis was performed. Patients were separated into different groups according to sex, age, clinical stage, histological grade, anatomic subdivision, alcohol history, smoking status, HPV status, and tumor mutation burden (TMB) value. These clinicopathological characteristics and the risk score served as covariates and ROC curves were also plotted to evaluate the value in predicting prognosis. Kaplan–Meier survival analysis was also performed in the testing sets from GEO and ICGC cohorts to validate the efficacy of our risk signature. *P* value < 0.05 was regarded as significant.

## 3. Results

### 3.1. Differentially Expressed Autophagy-Related Genes

**Figure 1:**
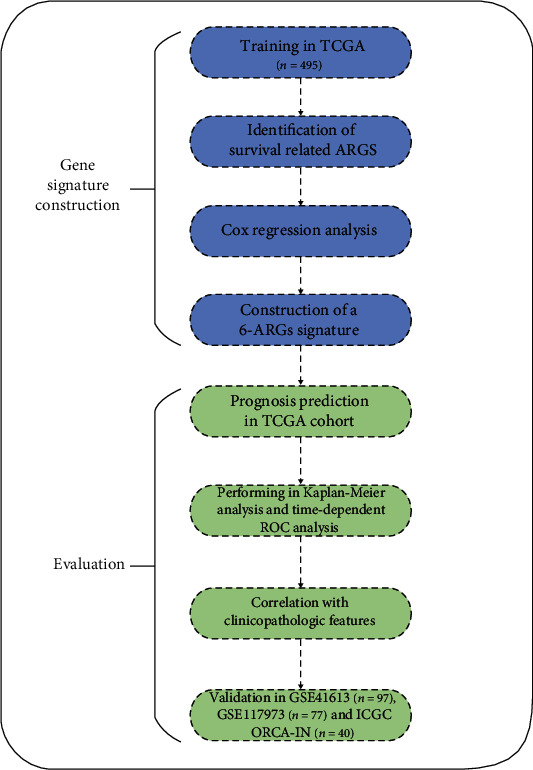
A schematic flowchart of this study.

**Figure 2:**
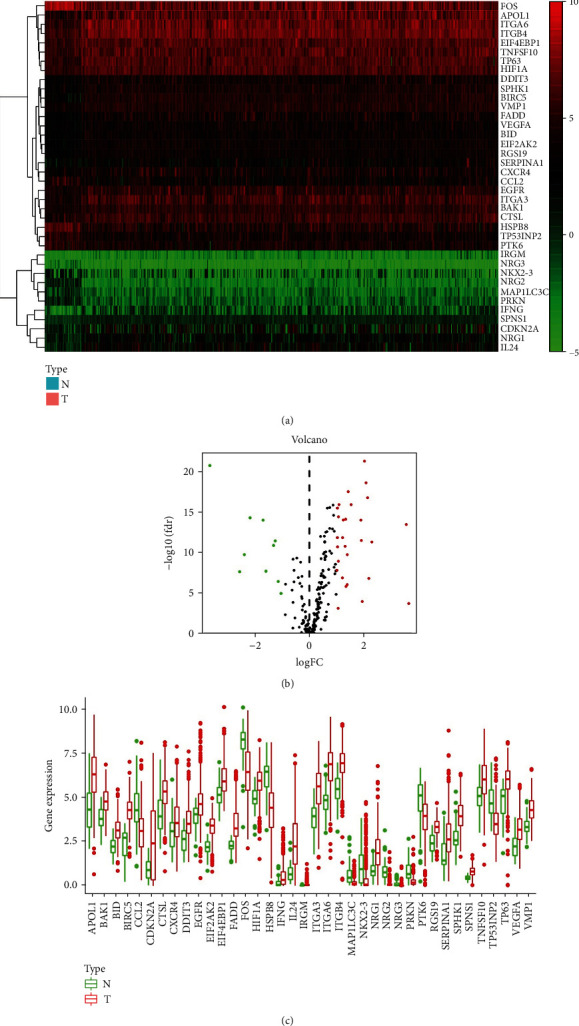
Differentially expressed autophagy-related genes (ARGs). (a) The heat maps of the 38 differently expressed ARGs. The red color indicates high gene expression while the green color indicates low gene expression. N indicates nontumor tissues; T indicates tumor tissues. (b) The volcano plot for the 232 ARGs from TCGA database. Red indicates high expression, and green indicates low expression. Black indicates that those genes show no difference between HNSCC and paired nontumor tissues. (c) The boxplot of the differentially expressed ARGs.

A flowchart was provided to demonstrate the study design and analysis (Figure 1). The RNA-seq profiles obtained from public database TCGA consisted of 501 HNSCC tissue samples and 40 nontumor samples. After extracting the expression data of 232 autophagy-related genes in HNSCC patients,10 downregulated genes (NRG2, NRG3, MAP1LC3C, PRKN, HSPB8, CCL2, FOS, TP53INP2, PTK6, and NKX2-3) and 28 upregulated genes (EIF4EBP1, BAK1, RGS19, HIF1A, CXCR4, CTSL, VMP1, SPNS1, TNFSF10, TP63, BID, VEGFA, SPHK1, EGFR, SERPINA1, DDIT3, EIF2AK2, ITGB4, ITGA3, APOL1, IRGM, BIRC5, FADD, ITGA6, IFNG, NRG1, IL24, and CDKN2A) were determined. Scatter plots displayed the expression patterns of differentially expressed ARGs between tumor and nontumor tissues (Figure 2).

### 3.2. Functional Enrichment Analysis of the Differentially Expressed ARGs

**Figure 3:**
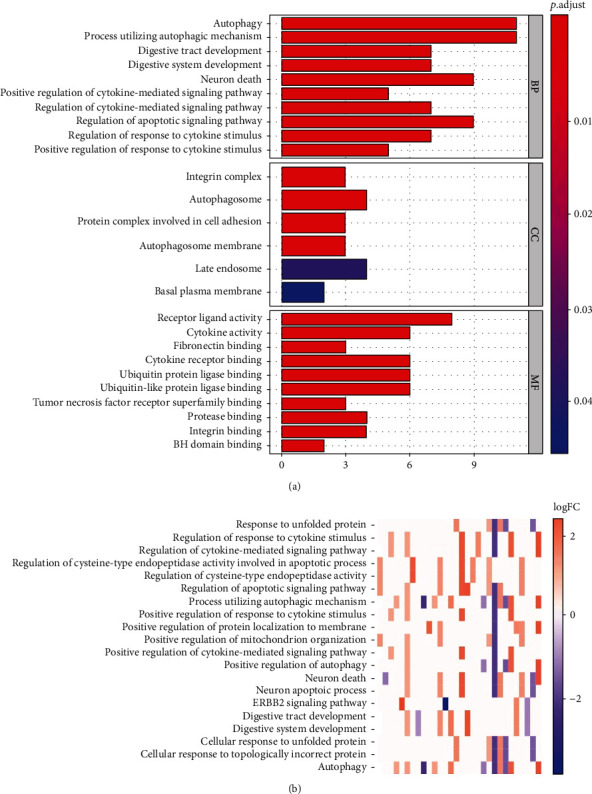
The barplot and heat map of gene ontology (GO) enrichment analysis. (a) The relationship between enriched GO terms and differentially expressed autophagy-related genes. BP indicates biological process; CC indicates cellular component; MF indicates molecular function. (b) The color of each block depends on the logFC value of the specified gene.

**Figure 4:**
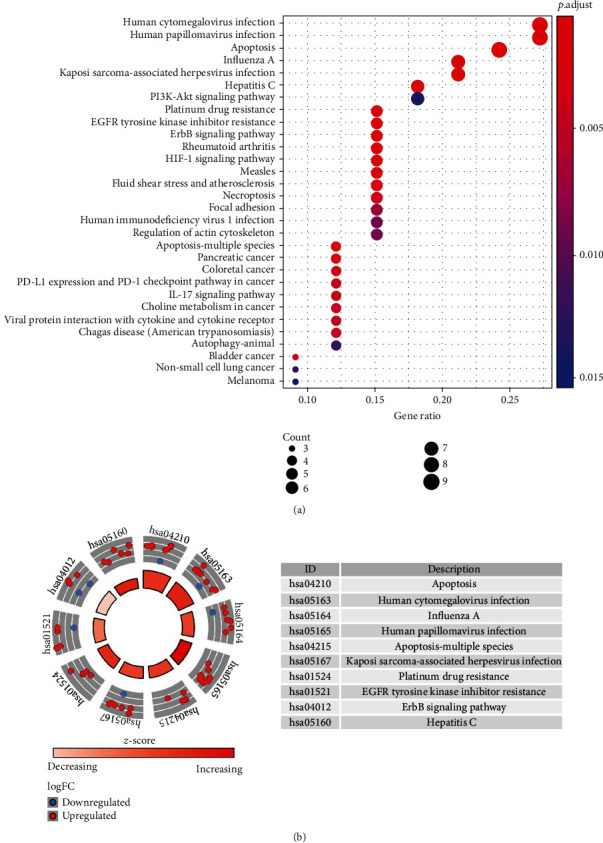
The bubble plot and circle plot of Kyoto Encyclopedia of Genes and Genomes (KEGG) pathway analysis. (a) The relationship between KEGG pathways and differentially expressed autophagy-related genes. (b) The outer circle shows a scatter plot for each term of the logFC value of the specified gene. Red circles display upregulation, and blue means downregulation. The higher the *Z*-score indicates, the higher expression of the enriched pathway.

Functional enrichment analysis was conducted to provide an in-depth understanding of biological functions of the 38 differentially expressed ARGs. The GO term functional enrichment and the KEGG pathway enrichment of these genes are summarized in Figures 3 and 4. The top enriched GO terms for biological processes were autophagy, process utilizing autophagic mechanism, neuron death, and regulation of apoptotic signaling pathway. Cellular components had integrin complex, autophagosome, protein complex involved in cell adhesion, and autophagosome membrane. Based on molecular function, genes were mostly enriched in terms of receptor ligand activity, cytokine activity, cytokine receptor binding, ubiquitin protein ligase binding, and ubiquitin-like protein ligase binding (Figure 3). The KEGG pathway enrichment analysis revealed that these genes were notably associated with pathways in apoptosis, human cytomegalovirus infection, and human papillomavirus infection. Most of the *Z*-scores of enriched pathways were more than zero, indicating that most of the pathways were more likely to be enhanced (Figure 4).

### 3.3. Identification of an Autophagy-Related Gene Signature for the Prognosis of HNSCC

A total of 495 eligible HNSCC patients based on TCGA database were involved in this study, and the baseline of clinical characteristics was presented (Table 1). To investigate differentially expressed ARGs for clinical prognosis, univariate Cox regression analysis was performed and 9 ARGs with *p* value < 0.05 were selected from the training set (Table 2). The candidate genes were passed on for LASSO regression. However, none of them were removed, suggesting that all 9 ARGs may provide specific information for prognosis (Figure S1). Subsequently, a multivariate Cox analysis was conducted, and six genes were identified to develop the prognostic signature (Table 3). The prognostic model was established based on 6 ARG expression levels and coefficients using the following formula: prognosis index (PI) = (0.0017∗EGFR) + (−0.0067∗HSPB8) + (0.6109∗PRKN) + (−0.0144∗CDKN2A) + (0.0075∗FADD) + (0.0046∗ITGA3).

**Figure 5:**
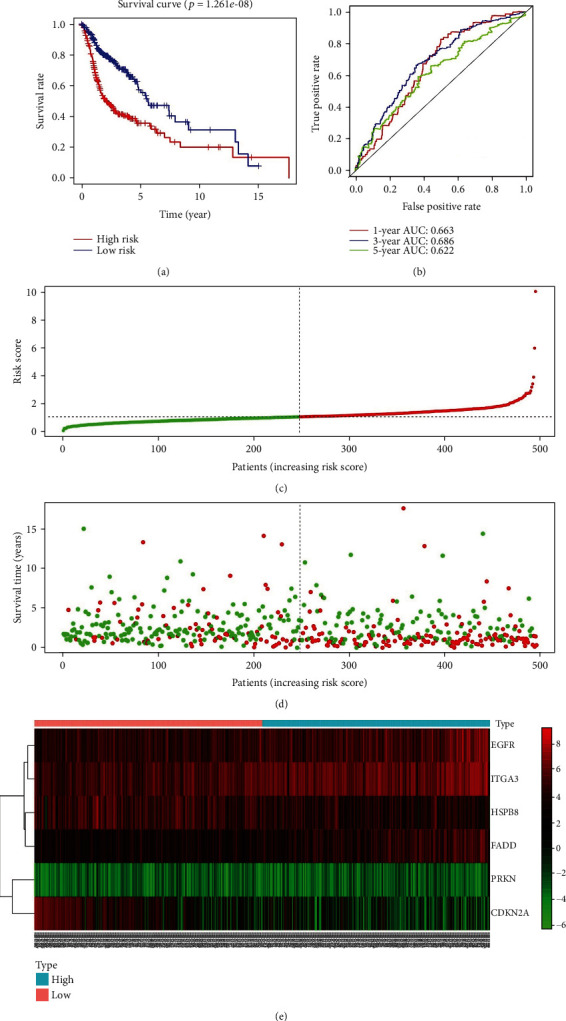
(a) The Kaplan–Meier plot of the overall survival (OS) for high-risk and low-risk patient cohorts divided by the 6-ARGs signature in the training set (*N* = 495). The OS differences are determined by the two-sided log-rank test. (b) Receiver operating characteristic analysis for the 6-ARG signature in predicting the patients of 1, 3, and 5 years OS in the training set. (c) The distribution of the risk score. Each dot represents a specific patient. The color red indicates high-risk level, and green indicates low-risk level. (d) The distribution of patients' survival status. Each dot represents a specific patient. The color red indicates dead, and green indicates alive. (e) The heat map displays the relationship between risk score and respective gene expression level of this signature in the training set.

**Figure 6:**
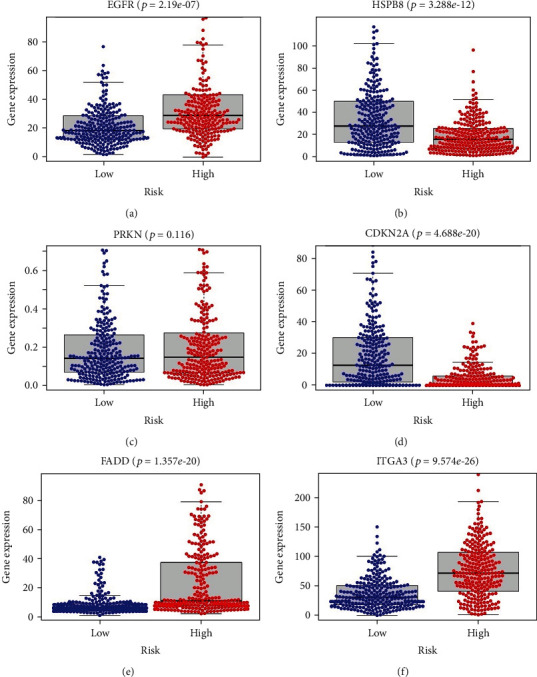
Different expression of the six key genes between the high-risk group and low-risk group.

**Figure 7:**
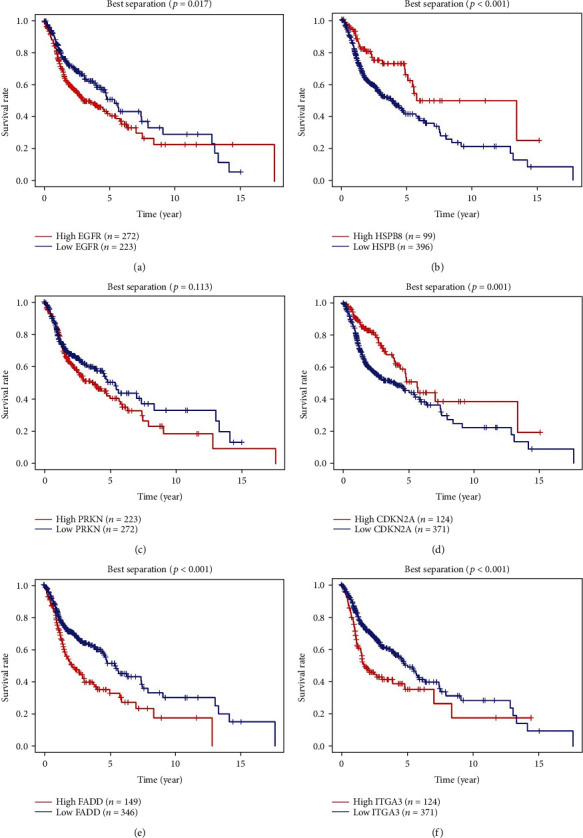
The correlation between the six genes included in the signature and HNSCC patients' overall survival. Kaplan–Meier plots demonstrate results from the analysis of correlation between each gene expression level and OS, all using the best separation.

Based on the median expression value of PI, patients in the training cohort were stratified into the high-risk and low-risk groups with specific cut-off point at 1.023. Survival analysis indicated that patients in the high-risk group had a significantly worse prognosis (Figure 5(a)). The results of ROC analysis revealed that this PI model had good accuracy and efficiency in predicting the 1-, 3-, and 5-year survival with respective areas under the ROC curve (AUCs) which were 0.663, 0.686, and 0.622 (Figure 5(b)). With an increasing risk score came shorter overall survival and more death events (Figures 5(c) and 5(d)). The heat map was also performed to evaluate the relationship between risk score and respective gene expression level in this signature (Figure 5(e)). EGFR, FADD, and ITGA3 were upregulated in the high-risk group (Figures 6(a), 6(e), and 6(f)), which indicated they acted as risk factors, while HSPB8 and CDKN2A were discovered to be downregulated in the high-risk group (Figures 6(b) and 6(d)), indicating that they were protective factors for HNSCC. However, we did not observe a significant difference of PRKN expression between the high-risk and low-risk groups (Figure 6(c)). Kaplan–Meier survival analysis of the 6 ARGs demonstrated similar results. The high expression of EGFR, FADD, and ITGA3 was significantly correlated with inferior overall survival of HNSCC patients (Figures 7(a), 7(e), and 7(f)). In contrast, the downregulation of HSPB8 and CDKN2A indicated inferior OS (Figures 7(b) and 7(d)). Also, there was no significant difference in OS concerning PRKN expression level (Figure 7(c)). Since each gene in this risk model played a different role in predicting prognosis, there may not be of statistical difference for every single gene in above analysis.

### 3.4. Correlation between the Autophagy-Related Gene Signature and Clinicopathologic Characteristics in HNSCC Patients

**Figure 8:**
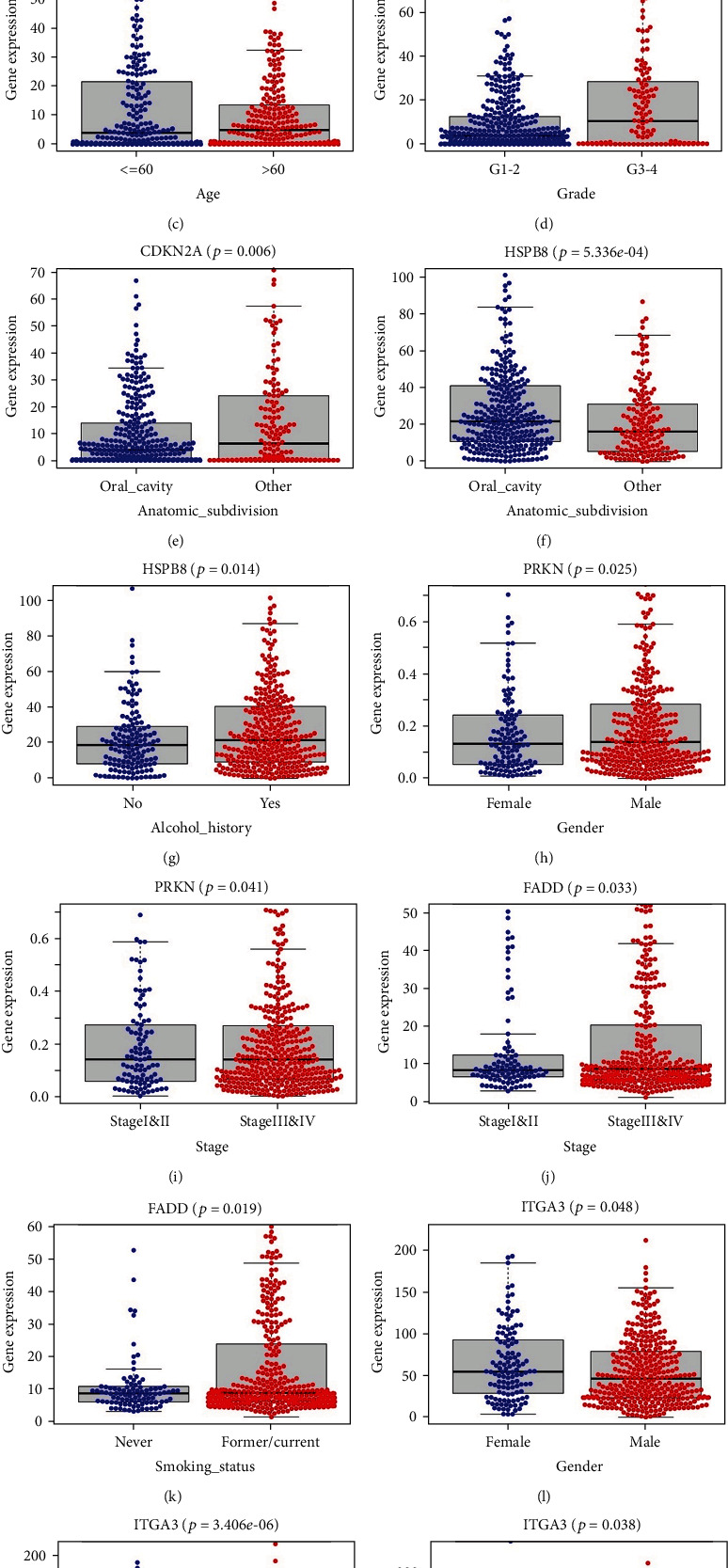
(a) The 6-ARG signature in the cohort stratified by clinical stages. (b–e) CDKN2A expression in the cohorts stratified by sexes, ages, grades, and anatomic subdivisions. (f, g) HSPB8 expression in the cohorts stratified by anatomic sites and alcohol histories. (h, i) PRKN expression in the cohorts stratified by sexes and clinical stages. (j, k) FADD expression in the cohorts stratified by clinical stages and smoking statuses. (l–n) ITGA3 expression in the cohorts stratified by sexes, anatomic subdivisions, and smoking statuses.

**Figure 9:**
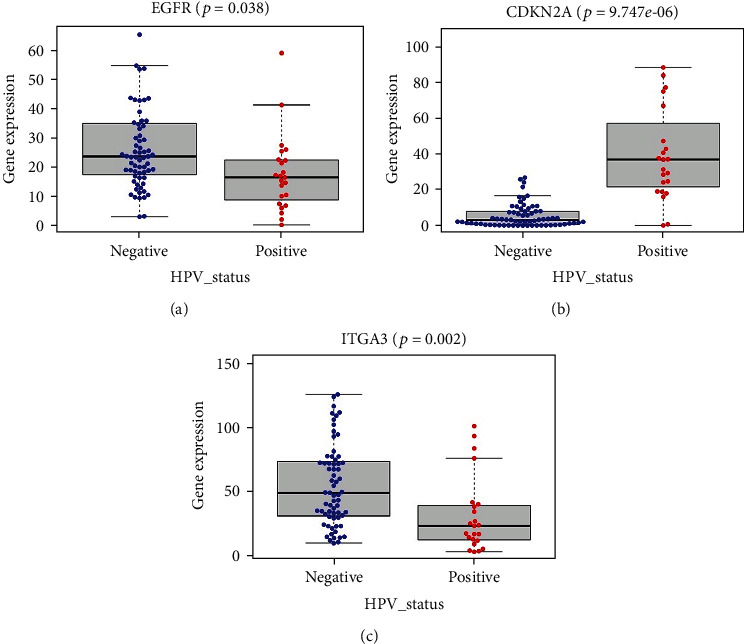
(a) EGFR expression in the cohorts stratified by HPV status. (b) CDKN2A expression in the cohorts stratified by HPV status. (c) ITGA3 expression in the cohorts stratified by HPV status.

Considering that clinical parameters may influence the performance of the risk signature, the patients were also grouped by sex, age, clinical stage, histological grade, anatomic subdivision, alcohol history, and smoking status and to explore their association with the 6-ARG signature. Patients with incomplete grade, alcohol history, and smoking information were removed, and 457 samples were retained for this procedure. The results suggested that the signature was significantly correlated with clinical stage (*p* = 0.037, Figure 8(a)). Besides, the 6 ARGs displayed different expression with respect to various clinicopathologic features. Different expression of CDKN2A was found across different sexes, ages, grades, and anatomic subdivisions (Figures 8(b)–8(e)). HSPB8 was expressed differentially with different anatomic sites and alcohol histories (Figures 8(f) and 8(g)). Higher PRKN expression was observed in male and stage III and IV HNSCC patients (Figures 8(h) and 8(i)), and higher FADD expression was found in patients with higher clinical stages and tobacco use (Figures 8(j) and 8(k)). ITGA3 also showed different expression across different sexes, anatomic subdivisions, and smoking statuses (Figures 8(l)–8(n)). Since HPV has been revealed to have some impacts on tumor progression in HNSCC [11], we also took HPV status into analysis. Because many patients in TCGA cohort were lack of HPV status information, only 90 samples were selected. As shown in Figure 9, higher expression levels of EGFR and ITGA3 were discovered in patients with HPV-negative HNSCC, while higher CDKN2A expression was found in patients with HPV-positive HNSCC.

### 3.5. The Autophagy-Related Gene Signature Is an Independent Prognostic Indicator in Patients with HNSCC

**Figure 10:**
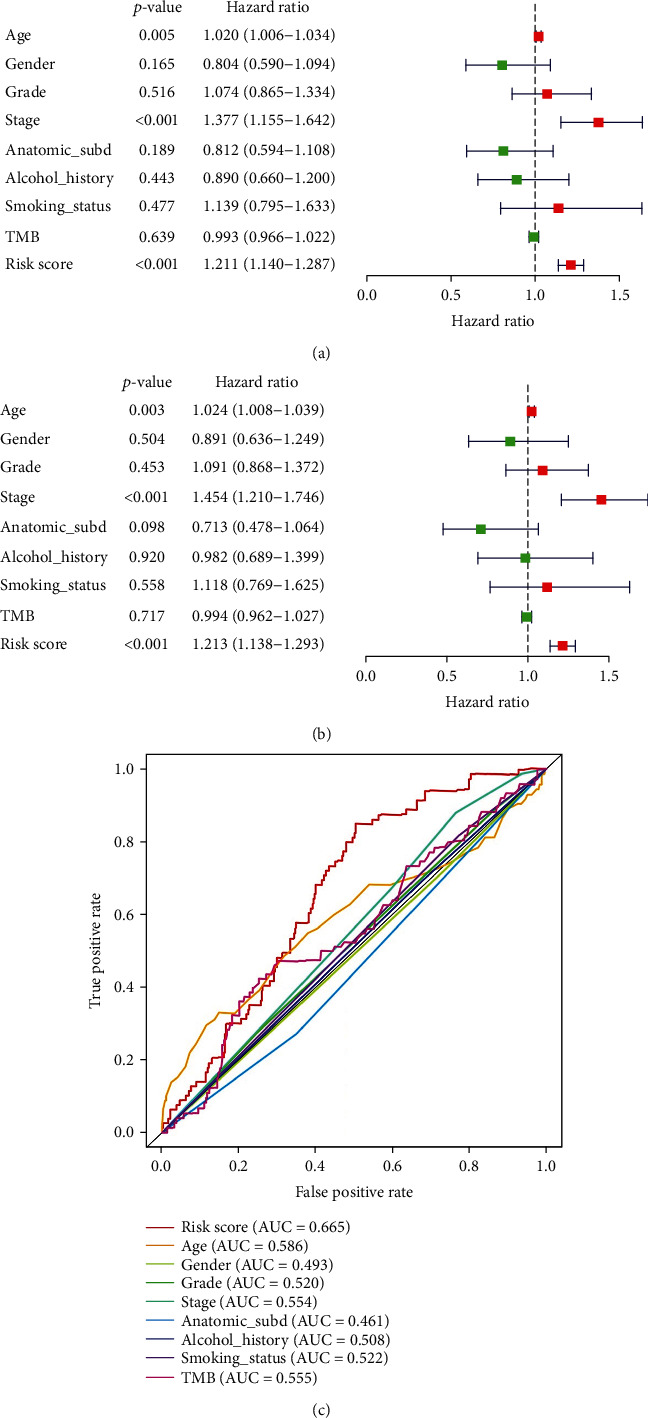
The forest plots of univariate (a) and multivariate (b) Cox analyses display the correlation of different indexes and overall survival of HNSCC patients. (c) Receiver operating characteristic analysis for risk signature and clinicopathological features in predicting HNSCC patients' OS in TCGA cohort.

**Figure 11:**
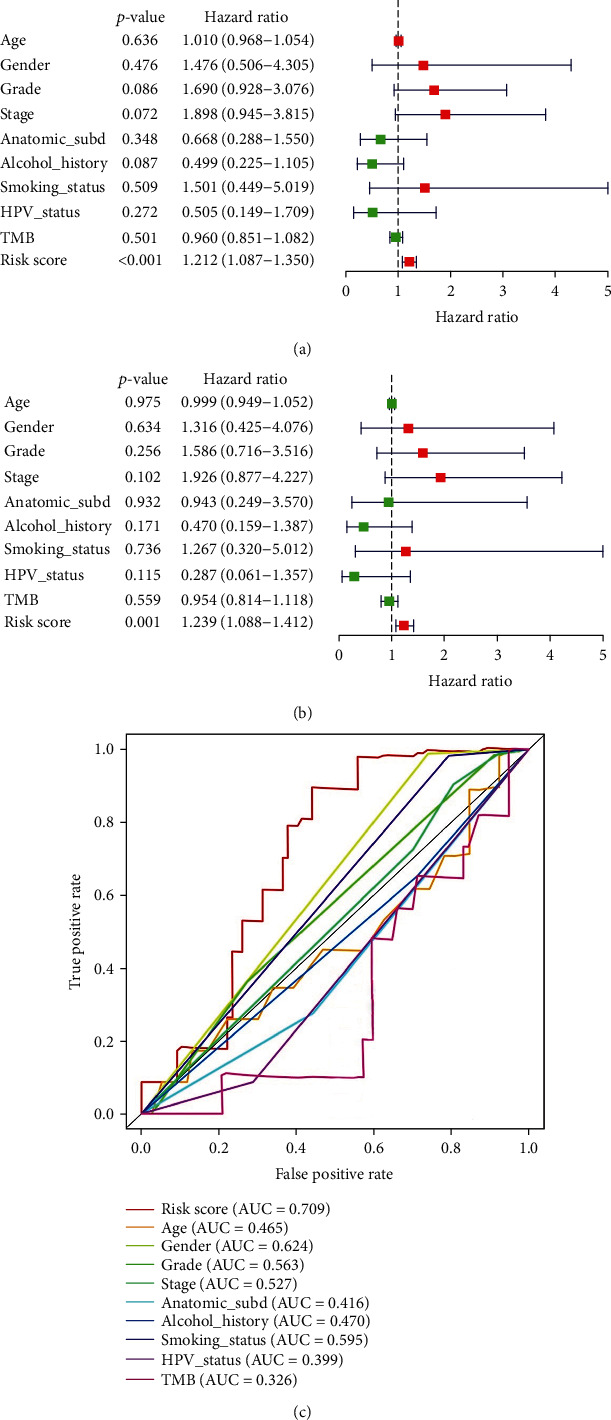
The forest plots of univariate (a) and multivariate (b) Cox analyses display the correlation of different indexes and overall survival of HNSCC patients with available HPV status information. (c) Receiver operating characteristic analysis for risk signature and clinicopathological features in predicting HNSCC patients' OS.

**Figure 12:**
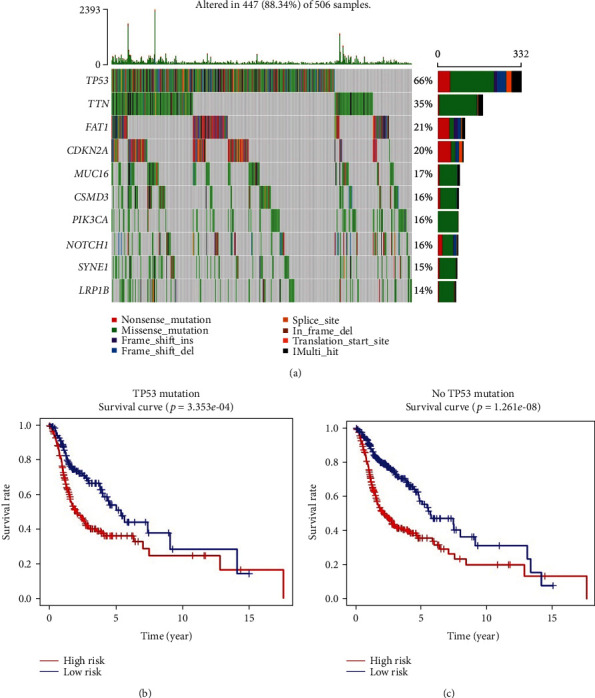
(a) Top ten genes with the highest mutation rates in HNSCC patients from TCGA database. (b) The results of Kaplan–Meier analysis in HNSCC patients with TP53 mutation. (c) The results of Kaplan–Meier analysis in HNSCC patients without TP53 mutation. The OS differences are determined by the two-sided log-rank test.

To identify other possible contributors on overall survival, the patients in different clinical subgroups were used for univariate and multivariate Cox regression analyses. Tumor mutation burden (TMB) has been shown to correlate with cancer aggressiveness and immunotherapeutic response [12, 13] and thus was also included in this procedure. 450 HNSCC patients with available TMB information were stratified in the high/low TMB group based on median TMB value and were selected for further study. The results suggested that the autophagy-related gene signature could independently predict prognosis of HNSCC (Figures 10(a) and 10(b)). It was also noticed that age and clinical stage were also significantly associated with patient survival. However, ROC analysis demonstrated that age (AUC = 0.586) and stage (AUC = 0.554) alone were not reliable indicators while risk score signature (AUC = 0.665) kept a stable and reliable performance (Figure 10(c)). Likewise, we brought HPV status into Cox regression analysis and 88 samples were selected. The results also indicated that the risk signature was an independent prognostic factor for HNSCC patients with even better predicting accuracy (AUC = 0.709, Figure 11). Besides, we identified the most frequently mutated genes in patients with HNSCC (Figure 12(a)). Researches have indicated TP53 to be the most commonly mutated gene in HNSCC [14, 15], which was also confirmed in our study. Since mutational status of the samples might influence the performance of the risk signature, the patients were grouped by mutational status of TP53. The high-risk groups showed significantly shorter OS in both HNSCC patients with TP53 mutation and patients without TP53 mutation (Figures 12(b) and 12(c)), indicating the independence of the risk signature once again. In conclusion, the 6-ARG signature could be applied as an independent prognostic indicator for HNSCC in clinical practice.

### 3.6. Validation of the Autophagy-Related Gene Signature in Independent HNSCC Cohorts

**Figure 13:**
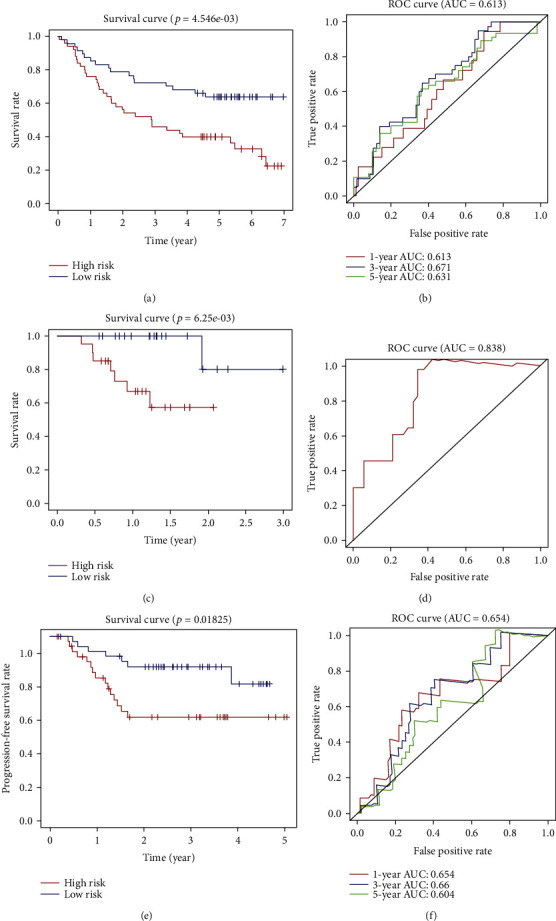
The Kaplan–Meier curves of the overall survival (OS) for high-risk and low-risk patient cohorts divided by the 6-ARG signature in the GEO dataset GSE41613 (a) and ICGC dataset ORCA-IN (c). (e) The Kaplan–Meier curves of the progression-free survival (PFS) for high-risk and low-risk patient cohorts divided by the 6-ARG signature in GSE117973. Receiver operating characteristic analysis in the GEO datasets GSE41613 (b) and GSE117973 (f) and the ICGC dataset ORCA-IN (d).

Three independent datasets GSE41613, GSE117973, and ORCA-IN (ICGC) were used as external validation groups. The risk score for each patient was calculated using the same PI formula. The patients were stratified into the high-risk and low-risk groups based on the median risk score. We performed Kaplan–Meier survival analysis in the three testing groups, which confirmed the prognostic value of our gene signature. As expected, patients in the high-risk groups from GSE41613 and ORCA-IN datasets showed significantly inferior overall survival (Figures 13(a) and 13(c)). The survival information of GSE117973 dataset was progression-free survival (PFS). Similarly, the high-risk HNSCC patients showed inferior PFS (Figure 13(e)). A good prognostic ability was also observed based on the time-dependent ROC analysis for the GSE41613 cohort in 1-, 3-, and 5-year follow-ups with respective AUC values which were 0.613, 0.671, and 0.631 (Figure 13(b)) and GSE117973 cohort in 1-, 3-, and 5-year follow-ups with respective AUC values which were 0.654, 0.66, and 0.604 (Figure 13(f)). Since there were only a limited number of samples acquired from ICGC and the follow-up information of most patients was restricted within 2 years, we just demonstrated the ROC curve of the 1-year follow-up (AUC = 0.838, Figure 13(d)), which suggested great predicting ability of this signature for HNSCC patients' prognosis once again. Therefore, the 6-ARG-based risk signature was proved to show great competence in predicting prognosis of HNSCC.

## 4. Discussion

HNSCC is one of the most prevailing and life-threatening cancers worldwide. Despite considerable improvements in diagnosis and treatment, the survival rate of HNSCC remains low. Besides, treatment is accompanied by significant long-term toxicity and morbidity [16]. Numerous studies have shown that autophagy plays a dual role in occurrence and progression of tumors. On one hand, autophagy promotes genomic stability and anticancer immunosurveillance in tumor suppression. On the other hand, it boosts high metabolic activity to sustain tumor cell survival and executives cytoprotective function under environmental stress in tumor progression [17]. Recent studies have indicated that various autophagy-related genes and corresponding protein expression profiles are related to clinicopathological features or prognosis of OSCC, including LC3, BECN1, ATG16L1, ATG9A, SQSTM1, and ATG5 [18]. However, most research mainly focuses on one particular gene related to autophagy.

The large-scale databases, such as TCGA and GEO, provide us with abundant information and effective measures to explore gene signatures. In this study, we screened autophagy-related genes and identified six key prognostic genes, which could be potential biomarkers and therapeutic targets. The 6-ARG signature was capable of distinguishing patients of different risk and prognosis, which was validated in multiple datasets and was proved to own great prognostic ability, thus providing us with a novel insight in HNSCC. The GO and KEGG analyses were performed to exploit the molecular and biological information of in-depth ARGs. The results of both functional analyses revealed that the top enriched terms were closely related to autophagy. It was also reported that autophagy contributed to membrane trafficking and signaling pathways in a diverse way [6]. Moreover, there was an increase of enriched KEGG pathways in human cytomegalovirus infection (HCMV) and human papillomavirus infection (HPV), indicating the interplay between autophagy and the immune microenvironment. Previous observations have indicated that HPV-associated HNSCC patients tended to demonstrate improved survival compared to patients with HPV-negative HNSCC. Integration of the HPV genome into the host's genome could generate remarkable downstream consequences in immune response, especially an enhanced infiltration of immune cells and inflammatory cytokines in the HPV-positive tumor microenvironment [19, 20]. This was consistent with our results, because the risk factors in the gene signature EGFR and ITGA3 were downregulated while the protective factor CDKN2A was upregulated in HPV-positive HNSCC patients. However, our study did not observe a significant correlation between HPV and prognosis of HNSCC patients. The reasons may be that HNSCC were a heterogeneous group of malignancies and HPV was detected in 25.9% of all HNSCC, mainly the oropharyngeal squamous cell cancer subtypes [21, 22].What is more, there were only a limited number of HNSCC patients equipped with available HPV status information from TCGA database that may also affect the results. HCMV was regarded as a factor for tumorigenesis and has been revealed to have connection with several cancers including malignant glioma, cervical carcinoma, Kaposi's sarcoma, and breast cancer [23]. Some of the HCMV gene products and proteins were proved to accelerate cancer progression via certain pathways, including suppression of the local immune response against tumors and promotion of cell apoptosis [24].

The Cox regression survival analysis helped us to identify six key prognostic ARGs and construct a prognostic signature, which could be an independent indicator for HNSCC patients' OS. We observed a decreasing expression level of *HSPB8* and *PRKN* and an increasing expression level of *EGFR*, *CDKN2A*, *FADD*, and *ITGA3* in tumor samples. Conversely and interestingly, *PRKN* was upregulated while *CDKN2A* was downregulated in the high-risk group. According to the results, we speculate that the performance of autophagy is not immutable and autophagy plays a complicated role in various kinds of tumors [25].

Indeed, four genes involved in this prognostic signature have been shown to be significantly correlated with carcinogenesis of HNSCC. The epidermal growth factor receptor (EGFR) is a cell surface receptor member of the ErbB family, which has been extensively studied in HNSCC. Activation of EGFR leads to the promotion of proliferation, differentiation, antiapoptotic signaling, angiogenesis, and metastatic spread and thus is highly correlated with tumor progression [26, 27]. EGFR is reported to be overexpressed in more than 90% of HNSCC and is an independent prognostic indicator which is associated with increased tumor size, shorter progression-free survival, and decreased overall survival [28–30]. This has made EGFR a promising therapeutic target and led to the development of monoclonal antibodies and tyrosine kinase inhibitors in anti-EGFR therapy [31]. Currently, the EGFR monoclonal antibody cetuximab is the only target drug that has been approved by the US FDA in the treatment for HNSCC [32].*CDKN2A*, a tumor-suppressor gene which encodes the cell cycle regulator p16INK4a, has been extensively investigated to be associated with HNSCC. Global genomic analyses suggest *CDKN2A* being one of the most commonly altered genes in HNSCC and noncoding mutations of *CDKN2A* correlates with worse overall survival in HNSCC patients [33, 34]. The results of immunohistochemistry suggest that oropharyngeal tumors with p16INK4a protein expression demonstrate better prognosis than p16-negative oropharyngeal tumors [35]. Meanwhile, HPV-positive patients with p16INK4a expression have better response to chemotherapy and radiotherapy compared with HPV-negative patients, during the standard multimodality treatments [36]. FADD is originally described as an adapter molecule mediating apoptosis, which has been newly revealed to engage in cell survival and tumor development [37, 38]. High FADD expression has been identified frequently in HNSCC (>30%) and is related to a higher incidence of lymph node metastasis and shorter distant metastasis-free internal [39]. Moreover, pharmacologic modulation of *FADD* is found to be effective and promising in HNSCC patients [40]. ITGA3 is a member of the integrin family and serves as a cell surface adhesion protein [41]. Research has demonstrated that knockdown of gene *ITGA3* inhibits HNSCC cancer cell migration and invasion. Furthermore, high expression of *ITGA3* indicates poorer survival of patients with HNSCC [42, 43].

So far, existing research also has revealed some mechanism of the other two identified ARGs, though their interaction with HNSCC is rudimentary and inconclusive. PRKN, also named as PARK2 or PARKIN, is an E3 ubiquitin ligase that functions in mitophagy and xenophagy. Biological studies demonstrate that *PRKN* possesses both prosurvival and growth suppressive capacities and its function as a tumor promoter or suppressor is highly dependent on cancer subtype and context [44, 45]. *PRKN* has been found to be affected in tumor microenvironmental signaling networks with likely loss of expression in HNSCC tumor samples [46]. Decreased expression of *PRKN* has also been identified in oropharyngeal squamous cell carcinoma (OPSCC) samples compared with paired normal tissues [47], which is consistent with our findings. But how *PRKN* behaves in tumor progression especially in HNSCC is still indefinite.

HSPB8 belongs to the ubiquitous small heat shock protein (sHSP) family, which cooperates with BAG3 and stimulate autophagic flux to seek out damaged cellular components for autophagy. Although there have been no reports concerning HNSCC and *HSPB8*, this gene plays an important role in some diseases. It is reported that mutation of *HSPB8* is linked to neurodegenerative disorders [48]. In addition, the decreased expression of HSPB8 has been shown to lead to an increased number of cells resting in the G0/G1 phase and reduce the migratory ability of MCF-7 cells and thus is engaged in regulating cell cycle and cell migration in MCF-7 cells of breast cancer [49]. Hence, the relationship between these prognostic ARGs and HNSCC needs further research in the future.

However, there are some limitations in this study. First, this is a retrospective study, which emphasizes on data mining and data analysis. Further experiments are needed to validate these findings. Second, there is currently no other research that investigates autophagy-related gene signature for HNSCC, suggesting that we are unable to validate our findings in another independent study. Therefore, we encourage multicenter data to confirm our results.

## 5. Conclusions

In summary, we identify a six-autophagy-related gene signature that can independently predict prognosis of HNSCC and help to distinguish high-risk patients. The six identified genes provide new insights into underlying molecular mechanisms of HNSCC and can be utilized as promising therapeutic targets. Further studies are expected to verify the clinical application and explore optimal treatment strategies for HNSCC.

## Figures and Tables

**Table 1:** Clinical characteristics of head and neck squamous cell carcinoma patients from TCGA in this study.

Features	Alive	Dead	Total
	(*n* = 281)	(*n* = 214)	(*n* = 495)
Gender			
Female	64	68	132
Male	217	146	363
Age			
Mean (SD)	59.44 (11.2)	62.97 (12.4)	60.97 (11.9)
Median [min, max]	60 [19, 85]	63 [24, 88]	61 [19, 88]
Anatomic subd			
Oral cavity	179	149	328
Other	102	65	167
Clinical stage			
Stage I	21	4	25
Stage II	50	30	80
Stage III	55	35	90
Stage IVA	150	134	284
Stage IVB	5	8	13
Stage IVC	0	3	3
Grade			
G1	39	22	61
G2	166	131	297
G3	63	53	116
G4	2	0	2
GX	9	7	16
NA	2	1	3
Alcohol history			
Yes	192	138	330
No	83	71	154
NA	6	5	11
Smoking status			
Never	67	42	109
Former/current	212	164	376
NA	2	8	10

SD: standard deviation; sub: subdivision.

**Table 2:** The results of univariate Cox analysis.

Gene	HR	HR.95L	HR.95H	*p* value
CXCR4	0.990886	0.98339	0.998439	0.018111
EGFR	1.001677	1.000173	1.003184	0.028858
HSPB8	0.993644	0.987676	0.999648	0.03803
PRKN	1.496081	1.114563	2.008194	0.007317
NKX2-3	0.889201	0.808232	0.978281	0.01592
CDKN2A	0.982237	0.97227	0.992306	0.000573
CTSL	1.004316	1.000584	1.008062	0.023384
FADD	1.011174	1.004614	1.017777	0.000819
ITGA3	1.004492	1.001521	1.007472	0.00302

HR: hazard ratio; L: low confidence interval; H: high confidence interval.

**Table 3:** The results of multivariate Cox analysis.

Gene	Coef	HR	HR.95L	HR.95H	*p* value
EGFR	0.0017	1.0017	1.0001	1.0033	0.0397
HSPB8	-0.0067	0.9933	0.9876	0.9991	0.0241
PRKN	0.6109	1.8421	1.4074	2.4109	0.0214
CDKN2A	-0.0144	0.9857	0.9754	0.9960	0.0066
FADD	0.0075	1.0075	1.0008	1.0142	0.0277
ITGA3	0.0046	1.0047	1.0016	1.0077	0.0027

Coef: coefficient; HR: hazard ratio; L: low confidence interval; H, high confidence interval.

## Data Availability

The data used to support the findings of this study are available from TCGA database (https://cancergenome.nih.gov/), the Gene Expression Omnibus database (https://www.ncbi.nlm.nih.gov/gds/), International Cancer Genome Consortium (https://dcc.icgc.org/releases/current/Projects/ORCA-IN), and the Human Autophagy Database (HADb; http://autophagy.lu/clustering/index.html).

## Supplementary Materials

Supplementary Materials Figure S1: the results of the least absolute shrinkage and selection operator regression. (a) The tuning parameter (lambda) selection in the least absolute shrinkage and selection operator (LASSO) model using 10-fold cross-validation via minimum criteria. Dotted vertical lines are drawn at the optimal values using the minimum criteria and the 1 standard error of the minimum criteria (the 1-SE criteria). (b) LASSO coefficient profiles of the 9-survival-related autophagy-related genes.
