# Supplementary material for: Prognostic Correlation of an Autophagy-Related Gene Signature in Patients with Head and Neck Squamous Cell Carcinoma
Source: Comput Math Methods Med. 2020 Dec 28;2020:7397132. doi: 10.1155/2020/7397132 (PMC7785385; doi:10.1155/2020/7397132)
Supplement: Supplementary Materials — Figure S1: the results of the least absolute shrinkage and selection operator regression. (a) The tuning parameter (lambda) selection in the least absolute shrinkage and selection operator (LASSO) model using 10-fold cross-validation via minimum criteria. Dotted vertical lines are drawn at the optimal values using the minimum criteria and the 1 standard error of the minimum criteria (the 1-SE criteria). (b) LASSO coefficient profiles of the 9-survival-related autophagy-related genes. [file 7397132.f1.doc]

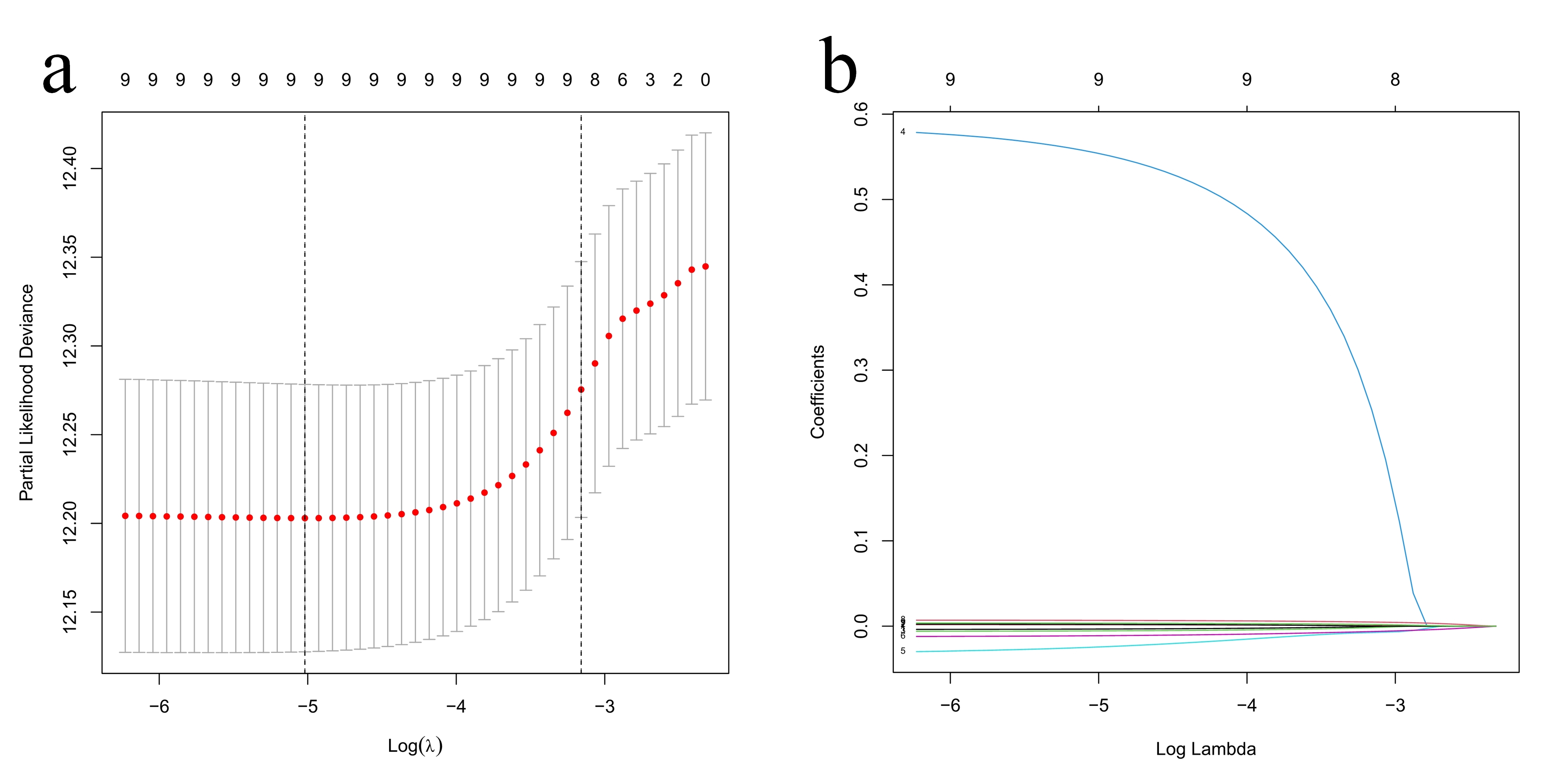


Figure S1 The results of the least absolute shrinkage and selection operator regression. (a) The tuning parameter (lambda) selection in the least absolute shrinkage and selection operator (LASSO) model using 10‐fold cross‐validation via minimum criteria. Dotted vertical lines are drawn at the optimal values using the minimum criteria and the 1 standard error of the minimum criteria (the 1‐SE criteria). (b) LASSO coefficient profiles of the 9‐survival related autophagy-related genes.
